# Editorial: New advances in biomedical research on sex, gender & gender incongruence

**DOI:** 10.3389/fendo.2025.1661856

**Published:** 2025-07-23

**Authors:** Sarah M. Burke, Rosa Fernández

**Affiliations:** ^1^ Department of Psychiatry, University Medical Centre Groningen, Groningen, Netherlands; ^2^ CICA - Interdisciplinary Centre for Chemistry and Biology Institute, Department of Psychology, University of A Coruña, A Coruña, Spain

**Keywords:** gender incongruence, gender identity, GAHT, AMAB: assigned male at birth, AFAB: assigned female at birth

The biomedical research landscape on sex, gender, and gender incongruence is evolving rapidly, reflecting growing awareness of the complex interplay between biological, psychological, and social dimensions that shape human identity. This Research Topic highlights the expanding scope of current investigations. These studies offer novel insights into the physiological, neurological, and psychological aspects of gender identity and gender-affirming care. Together, they underscore the importance of a multidisciplinary approach to advancing research on gender incongruence research.

A significant theme emerging from this Research Topic is the profound impact of gender-affirming hormone therapy (GAHT) on various physiological systems. Tienforti et al. conducted a meta-analysis on the effects of testosterone-based GAHT on renal function in transgender individuals assigned female at birth (AFAB). Their findings indicate a transient decrease in estimated glomerular filtration rate (eGFR), an index of renal functioning, during the initial year of therapy, which subsequently stabilises. Crucially, this change is attributed to an increase in creatinine production resulting from muscle mass gain, rather than actual kidney damage, highlighting the need for careful interpretation of eGFR in this population and advocating for sex-independent assessment tools. Complementing this, Ceolin et al. investigated body composition and perceived stress levels in transgender individuals after one year of GAHT. They found that AFAB individuals receiving testosterone attained bone mineral density levels comparable to those of cisgender individuals, assigned male at birth (AMAB), along with notable increases in muscle strength. Conversely, AMAB individuals receiving oestrogen-based GAHT exhibited increased fat mass and decreased lean mass, while their bone mineral density remained lower than that of AFAB cisgender controls. Significantly, perceived stress levels remained elevated in transgender individuals even after one year of GAHT.

Beyond somatic changes, the neurobiological underpinnings of gender identity and the effects of GAHT on brain structure are also a critical area of inquiry. Kim et al. explored differences in subcortical volumes between AFAB transgender individuals undergoing testosterone-based GAHT and a group of premenopausal AFAB cisgender individuals. Their study revealed significantly larger grey matter volumes in specific brain regions, including the caudate nucleus, hypothalamus, thalamus, and the right hippocampal subiculum. In the transgender group these volumetric changes positively correlated with free-testosterone levels, suggesting neuroplastic responses to hormone therapy and contributing to our understanding of the brain’s adaptability to hormonal environments. Delving deeper into neurodevelopment, Tawata et al. investigated how early-life reductions in sex steroids relate to atypical neurodevelopment, and differences in sensory sensitivity in individuals with Klinefelter syndrome and AMAB sexual minorities. Their work challenges the “Extreme Male Brain” theory, by suggesting that diminished, rather than excessive androgen action may contribute to certain neurodevelopmental atypicalities, underscoring a more complex influence of early hormonal environments on brain development and sensory processing.

The psychological well-being of transgender individuals and the tools to assess it are equally vital. Cortez et al. provided a comprehensive assessment of the sociodemographic and psychiatric characteristics of transgender adults attending a large Midwest tertiary medical centre. Their findings revealed a high prevalence of mental health disorders, including depression, anxiety, and ADHD, and a concerning rate of suicide attempts. This study underscores the urgent need to integrate mental healthcare into gender-affirming services, irrespective of sociodemographic factors. Möck et al. developed and validated the Kiel Gender Dysphoria Questionnaire to enable more precise, longitudinal tracking of psychological distress. Developed to assess distress related to gender incongruence in adults, this tool demonstrates strong psychometric properties, offering clinicians and researchers a reliable instrument for monitoring gender dysphoria over time.

In conclusion, this Research Topic represents significant advances in biomedical research on sex, gender, and gender incongruence. From elucidating the physiological and neurological impacts of GAHT to addressing the critical mental health needs and developing refined assessment tools, these studies collectively demonstrate the multifaceted nature of gender identity and the gender affirmation process. They underscore the need for sustained interdisciplinary research that integrates insights from endocrinology, neuroscience, psychology, and public health.

